# Malignant brachial plexopathy: A pictorial essay of MRI findings

**DOI:** 10.4103/0971-3026.73543

**Published:** 2010-11

**Authors:** Veena R Iyer, Darshana A Sanghvi, Nikhil Merchant

**Affiliations:** 1 Department of Radiology, Seth G. S. Medical College and King Edward Memorial Hospital, Mumbai – 400 012, India; 2 Department of Radiology, Kokilaben Dhirubhai Ambani Hospital, Mumbai – 400 053, India; 3 Department of Radiology, Tata Memorial Hospital, Mumbai – 400 012, India

**Keywords:** Brachial plexus, malignant peripheral nerve sheath tumor, MRI, radiation-induced plexopathy

## Abstract

For imaging, the brachial plexus is a technically and anatomically challenging region of the peripheral nervous system. MRI has a central role in the identification and accurate characterization of malignant lesions arising here, as also in defining their extent and the status of the adjacent structures. The purpose of this pictorial essay is to describe the MRI features of primary and secondary malignant brachial plexopathies and radiation-induced brachial nerve damage.

## Introduction

Primary and secondary tumors are the most common causes of brachial plexopathies in adults.[1] Metastatic breast and lung cancer are the most common nontraumatic causes of brachial plexopathy, after radiation fibrosis.[2] Malignant transformation of peripheral neurofibromas is the leading cause of death in patients with neurofibromatosis-1.[3] MRI, with its superior soft tissue discrimination and multi-planar imaging capabilities, is of special value in the characterization of tumors of the brachial plexus, with the added advantage that it allows visualization of the growth of these tumors along the axis of the nerve. In this pictorial essay we review the anatomy of the brachial plexus, discuss the clinical presentation of malignant brachial plexopathies, and illustrate their characteristic MRI features. MRI is also the technique of choice to distinguish between radiation-induced nerve damage and tumor recurrence; the features of radiation-induced plexopathy are described in this article.

## Anatomy

**Figure 1 (A– D):**
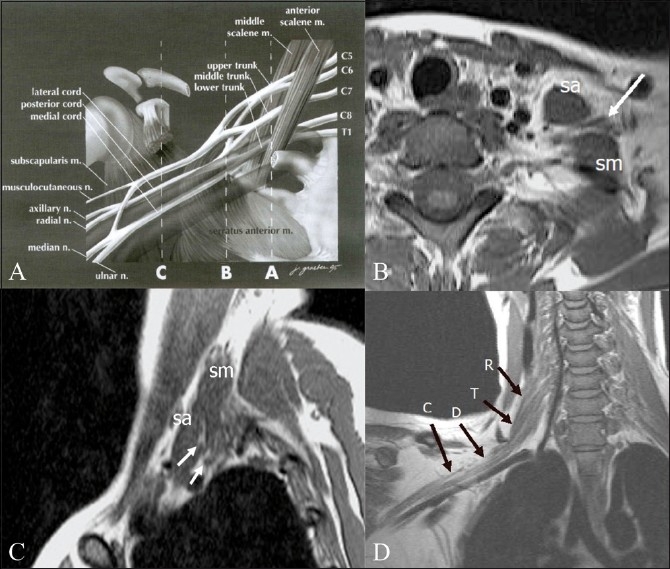
Diagrammatic representation (A) of the anatomy of the brachial plexus, with the musculoskeletal and vascular landmarks indicated by three interrupted lines: A — sagittal plane that intersects the interscalene triangle, B — sagittal plane through the lateral margin of the first rib, and C — sagittal plane of the coracoid process. (m, muscle; n, nerve). Axial (B) and sagittal (C), T1W images of the plexus in the interscalene triangle. The anterior and middle scalene muscles are the boundaries of the interscalene triangle and act as useful radiological landmarks for locating the roots and trunks (arrows) of the brachial plexus that lie within the triangle. (sa: scalenus anterior; sm: scalenus medius.) Oblique coronal T1W image (D) shows the roots (R), trunks (T), divisions (D), and cords (C) of the right brachial plexus. A — Reprinted from Neuroimaging Clinics of North America, 14(1), Bowen BC, Pattany PM, Saraf-Lavi E, and Maravilla KR, The brachial plexus: normal anatomy, pathology, and MR imaging, page 27, Copyright 2004. With permission from Elsevier

**Figure 2:**
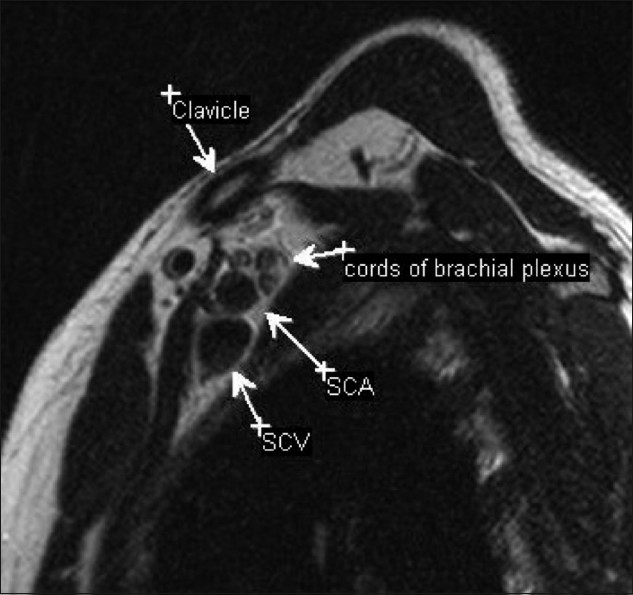
Sagittal T2W image through the axilla showing the relationship of the cords to the vessels. In the axilla, the three cords are identified posterosuperior to the vessels. (SCA: subclavian artery; SCV: subclavian vein)

**Figure 3 (A– C):**
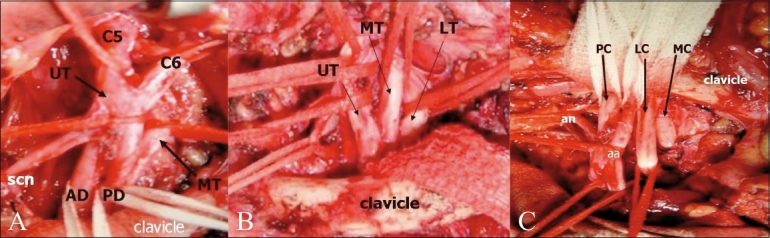
The surgical anatomy is based on the relationship of the brachial plexus to the clavicle. Intraoperative photographs show the supraclavicular (A), retroclavicular (B), and infraclavicular (C) portions of the brachial plexus. (Photograph courtesy: Ketan Desai, Department of Neurosurgery, Seth GS Medical College and KEM Hospital) (UT: upper trunk; MT: middle trunk; LT: lower trunk; AD: anterior division; PD: posterior division; scn: suprascapular nerve; PC: posterior cord; LC: lateral cord; MC: medial cord; an: axillary nerve, and aa: axillary artery)

The brachial plexus is formed by the anterior rami of the cervical nerves C5 – C8 and T1. The C4 nerve frequently gives a branch to C5, and T1 often receives a branch from T2. The roots exit the intervertebral foramina between the anterior and middle scalene muscles in the interscalene triangle [Figure 1]. As they exit the triangle formed by the scaleni, C5 and C6 form the upper trunk, while C8 and T1 form the lower trunk. The C7 nerve root continues as the middle trunk. As the three trunks pass under the clavicle, they divide into anterior and posterior divisions at the lateral edge of the first rib. In the axilla, the anterior divisions of the upper and middle trunk form the lateral cord, while the anterior division of the lower trunk continues as the medial cord. The posterior divisions of all three trunks unite to form the posterior cord. The cords are named based on their relationship to the second part of the axillary artery [Figure 2]. Individual branches arising from the cords provide motor and sensory innervations to the upper extremities. The surgical anatomy is based on the relationship of the brachial plexus to the clavicle [Figure 3]. The clavicle divides the plexus into supraclavicular, retroclavicular, and infraclavicular segments. The supraclavicular brachial plexus consists of roots and trunks. The divisions pass behind the clavicle, forming the retroclavicular plexus, while the cords lie in the infraclavicular segment. For radiologists, the anterior and middle scalene muscles are important landmarks for identifying the plexus; the roots and cords of the brachial plexus are located in the fascial plane, between the two muscles.

## Technique of MRI Scanning

The use of a body coil for imaging the brachial plexus provides a good field of view, although the signal-to-noise (SNR) ratio of the image is poor. Surface coils increase the SNR, but only over a small portion of the brachial plexus.[4]

The plexus is imaged effectively using a combination of the quadrature head coil, the first two elements of a phased-array spine coil, and a neck coil. T1W images display the regional anatomy, including muscles, vessels, and nerves outlined by fat planes. T2W sequences are useful in detecting pathological changes within the components of the plexus. Short tau inversion recovery (STIR) and frequency-selective fat saturation are both techniques used to null the signal from fat. Gadolinium contrast is useful in assessing malignant tumors as well as radiation injuries. T1W spin-echo images, with frequency-selective fat saturation are acquired immediately following contrast injection.

## Clinical Presentation

Malignant tumors in the plexus infiltrate the spinal column and neural foramina and result in mixed motor and sensory loss.[5] Radiation-induced fibrosis usually presents with sensory deficits. Neurological deficits, rapid increase in size of the mass, and severe pain at rest (that is not relieved by analgesics) in the distribution of a major nerve strongly suggest malignancy – a primary tumor of the brachial plexus.[6]

### MRI features of primary tumors

**Figure 4 (A,B):**
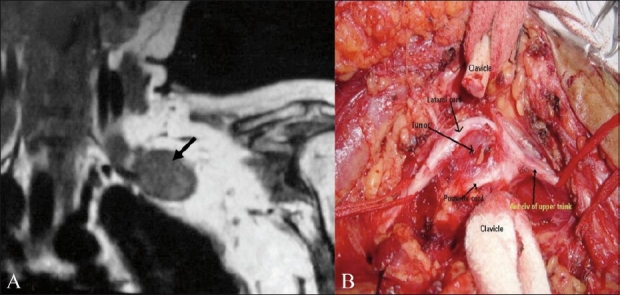
A 57-year-old male with histopathologically proven malignant schwannoma. Preoperative coronal T1W MRI (A) shows a well-marginated. low-signal-intensity solid mass (arrow) arising from the plexus. The corresponding intraoperative photograph (B) shows the tumor (arrow) seen after dividing the clavicle. The mass splays the lateral and posterior cords. (Photograph courtesy: Ketan Desai, Department of Neurosurgery, Seth GS Medical College and KEM Hospital)

**Figure 5 (A, B):**
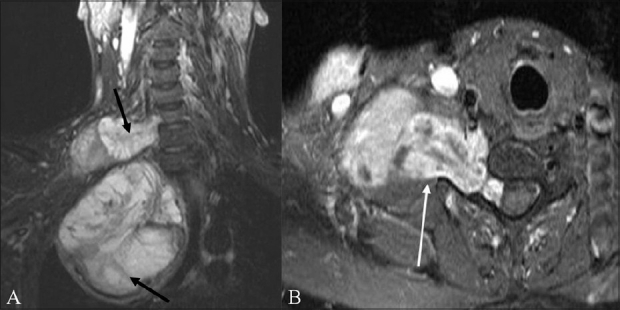
A 59-year-old male with neurofibrosarcoma involving the right brachial plexus. Coronal STIR (A) image shows a large heterogeneous, predominantly hyperintense, mass arising from the right brachial plexus (arrows). Focal linear low-signal septae are seen within. Contrast-enhanced, fat-suppressed, axial (B) image shows the mass enhancing intensely; it is dumbell-shaped, with a small intraspinal component that indents the thecal sac, and a large paravertebral component

Malignant peripheral nerve sheath tumor (MPNST) is also known as neurofibrosarcoma, neurogenic sarcoma, malignant neurofibroma, and malignant neurilemmoma [Figure 4]. About 3 – 13% of patients with neurofibromatosis-1, especially those who may have received radiotherapy, develop malignant transformation of a neurofibroma.[2] Although the MRI features of benign and malignant neural tumors overlap, a large tumor size, irregular margins with invasiveness, and a heterogenous appearance are findings that suggest malignancy. The lack of a ‘target sign’ (a hyperintense rim with centrally decreased intensity on T2W images, seen in neurofibromas) indicates that the classic findings of a benign neuroma are absent and this may be suggestive of malignancy [Figure 5]. On postcontrast imaging, malignant tumors predominantly show peripheral enhancement, with lack of central enhancement due to central necrosis and hemorrhage.[6] The role of MRI in these tumors is vital, to resolve important preoperative questions about occult proximal intraneural spread, intradural extension, and anatomical relationship with the vertebral arteries.[7]

### MRI features of malignant tumors involving the brachial plexus by direct spread

**Figure 6 (A, B):**
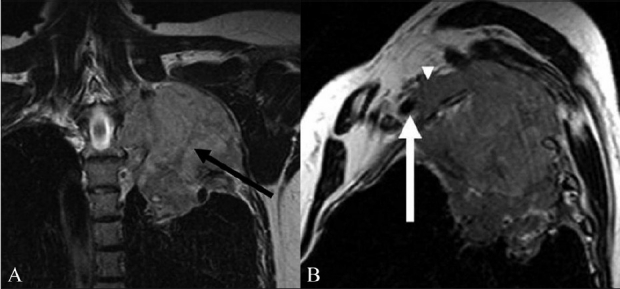
A 60-year-old male with Pancoast tumor. Coronal T2W (A) image shows a tumor of the apex of the left lung extending into the supraclavicular fossa to involve the trunks and divisions of the left brachial plexus (arrow). The mass is solid, with an intermediate T2 signal. The Sagittal T2W (B) image shows the anterosuperior component of the mass involving the divisions of the brachial plexus (arrowhead) posterosuperior to the subclavian vessels. The mass abuts the left subclavian artery (arrow)

**Figure 7 (A, B):**
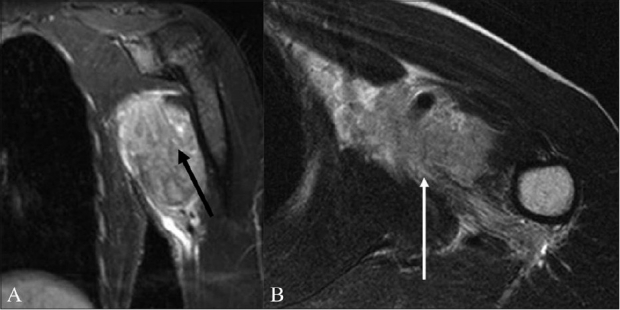
A 62-year-old male with a soft tissue sarcoma of the chest wall and axilla. The coronal STIR (A) and axial T2W (B) images show a hyperintense mass that infiltrates the cords of the brachial plexus with encasement of the axillary vessels (arrow)

When lung cancer arises in the superior pulmonary sulcus and is limited to the upper apical segments, but invades the para-apical structures, it is called a Pancoast tumor.[8] Pancoast tumors spread contiguously to involve segments of the brachial plexus [Figure 6]. Although the MRI features of Pancoast tumors are nonspecific, the use of fat-suppression and contrast provides good differentiation of the tumor from the surrounding normal tissue. Bone and soft tissue tumors arising in the adjacent structures also involve vessels and nerves in the axilla [Figure 7]. Osteosarcoma, aggressive fibromatosis, and Ewing sarcoma are some of the tumors that may cause brachial plexopathy. The MRI features resemble those of the primary tumor.

### MRI features of metastases that involve the brachial plexus

**Figure 8 (A, B):**
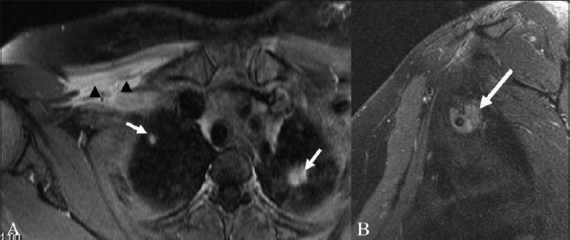
A 43-year-old female with metastatic breast cancer. Axial fat-suppressed, contrast-enhanced T1W (A) image shows abnormal enhancing soft tissue metastases along the right anterior chest wall, partially encasing the trunks of the right brachial plexus (arrowheads). Also seen in this image are bilateral enhancing pulmonary metastases (arrows). The abnormal perineural soft tissue (arrow) extends into the axilla, encasing the divisions and cords of the plexus, as seen on the sagittal fat-suppressed T1W image (B)

**Figure 9 (A, B):**
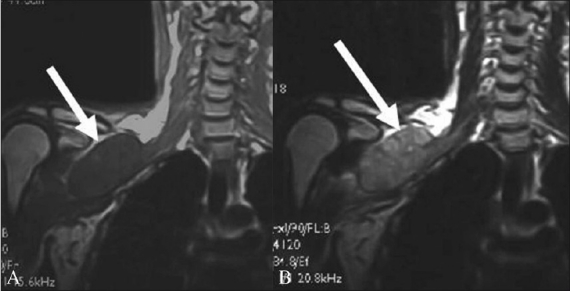
A 71-year-old male with buccal squamous cell carcinoma and supraclavicular lymph node metastasis. The coronal T1W (A) image shows a low-signal-intensity metastatic lymph nodal mass compressing the trunks, divisions, and cords of the right brachial plexus (arrow). On the corresponding coronal T2W (B) image, the mass shows high intensity (arrow)

**Figure 10 (A, B):**
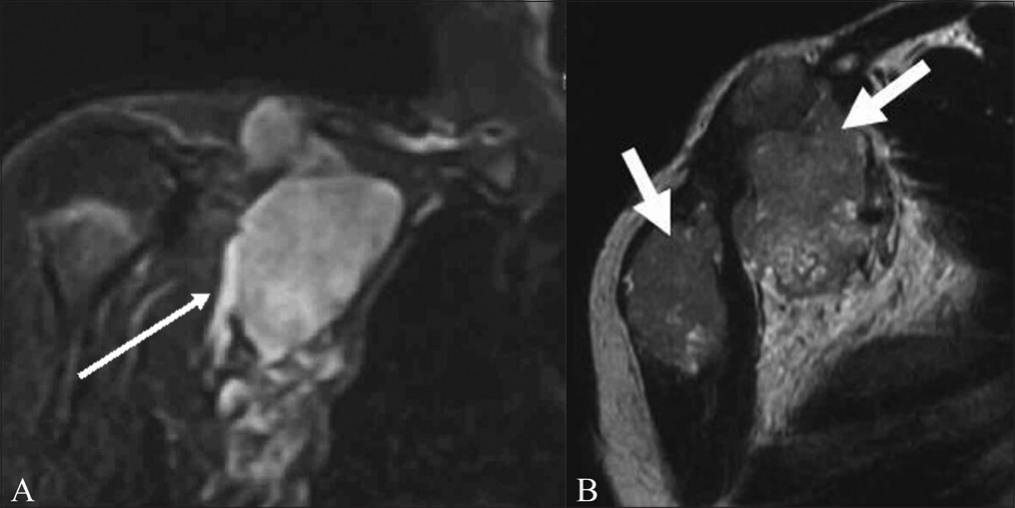
A 50-year-old-male with lymphoma. Coronal STIR (A) image shows a large multilobulated nodal mass involving the right brachial plexus (arrow). Sagittal T2W (B) image shows the lymph nodal mass encasing the divisions of the brachial plexus and the adjacent subclavian artery and vein (arrow)

**Figure 11 (A,B):**
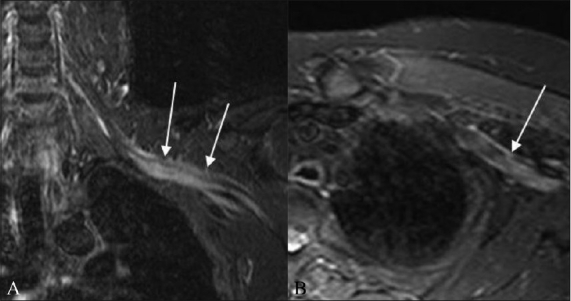
A 52-year-old-male with biopsy-proven neurolymphomatosis. Coronal (A) and axial (B) STIR images show thickening and increased signal in the left brachial plexus (arrows)

Secondary tumors involving the brachial plexus are more common than primary tumors. Breast and lung cancer are the most common cancers that involve the brachial plexus.[2] Lymphoma, melanoma, squamous cell carcinomas arising in the head and neck, and malignant mesotheliomas are some of the tumors that metastasize to the axillary nodes and involve the plexus. Typically, metastases from all causes have low signal intensity (SI) on T1W images and a high SI on T2W images, relative to the signal of muscle [Figures 8 and 9]. Metastases show enhancement on gadolinium-enhanced images. Occasionally they may demonstrate low SI on T2W images.[9] In cases of lymphoma, enlarged nodes can either directly compress the plexus or infiltrate it [Figure 10]. Neurolymphomatosis is a rare manifestation of lymphoma that predominantly involves the peripheral nerves and can involve the brachial plexus. MRI shows diffuse thickening of the plexus components, with marked T2 hyperintensity and postcontrast enhancement [Figure 11].

### MRI features of postradiation plexopathy and radiation fibrosis

**Figure 12 (A,B):**
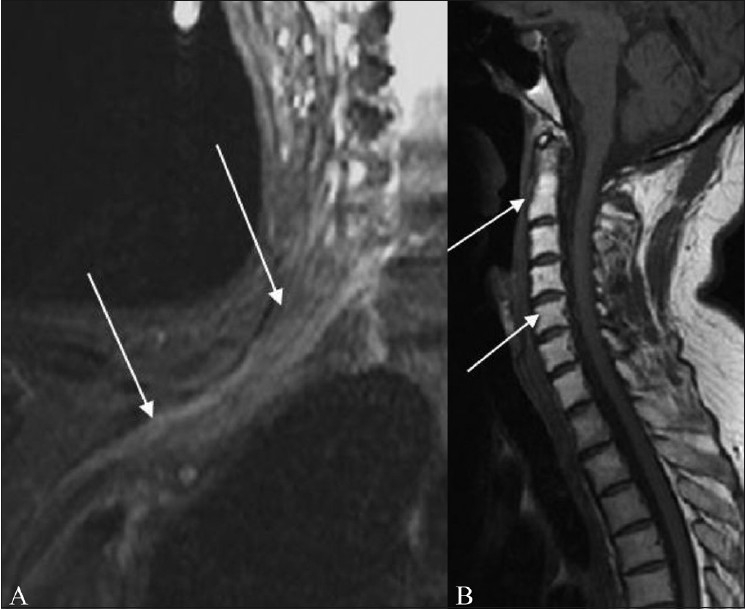
A 60-year-old female presenting with right upper limb weakness 18 months after radiation therapy for breast cancer. Coronal fat-suppressed T2W image (A) shows radiation plexopathy appearing as an abnormal thickening, and increased signal in the right brachial plexus (arrow). Sagittal T1W image (B) shows radiation-induced fat marrow replacement in the cervical vertebrae (arrows)

Radiation therapy to the neck and axilla may result in brachial plexopathy either due to radiation damage of the nerve or due to nerve compression by surrounding fibrous connective tissue. The median interval between irradiation and occurrence of brachial neuropathy has been reported to be one to four years, although neuropathy has been seen even 21 years after radiation therapy.[10] Two phases of neuropathy following irradiation have been described where early changes include electrophysiological and histochemical damage in the nerves themselves and the later phases include fibrosis of the soft tissue surrounding the nerves.[10] Clinical symptoms and electromyography cannot always differentiate a recurrence from brachial plexus injury, although painless upper trunk lesions with lymphedema suggest radiation injury, and painful lower trunk lesions with Horner syndrome are usually indicative of tumor infiltration.[11] MRI plays an important role in determining the diagnosis. Imaging features suggestive of radiation-induced plexopathy include diffuse, uniform, symmetric swelling, and T2 hyperintensity of the plexus within the radiation field.[12] Mild contrast enhancement may also be present, sometimes making differentiation from tumor difficult. Nonuniform, asymmetric, and focal enlargement, and the presence of a mass with postcontrast enhancement, usually indicate tumor recurrence. Radiation fibrosis often shows low SI on both T1W and T2W images.[12] T2 fat-suppressed and STIR sequences help to differentiate radiation fibrosis [Figure 12] from tumor.[2]

## Conclusion

A thorough knowledge of the complex anatomy of the plexus, the clinical features of patients presenting with malignant plexopathies, and the MRI features of these entities allow the radiologist to make the correct diagnosis.
